# TccP2-mediated subversion of actin dynamics by EPEC 2 – a distinct evolutionary lineage of enteropathogenic *Escherichia coli*

**DOI:** 10.1099/mic.0.2006/004325-0

**Published:** 2007-06

**Authors:** Andrew D. Whale, Rodrigo T. Hernandes, Tadasuke Ooka, Lothar Beutin, Stephanie Schüller, Junkal Garmendia, Lynette Crowther, Mônica A. M. Vieira, Yoshitoshi Ogura, Gladys Krause, Alan D. Phillips, Tania A. T. Gomes, Tetsuya Hayashi, Gad Frankel

**Affiliations:** 1 Division of Cell and Molecular Biology, Imperial College London, London SW7 2AZ, UK; 2 Departamento de Microbiologia, Imunologia e Parasitologia, Universidade Federal de São Paulo, São Paulo, Brazil; 3 Division of Bioenvironmental Science, Frontier Science Research Center, University of Miyazaki, 5200 Kiyotake, Miyazaki 889-1692, Japan; 4 Nationales Referenzlabor für *Escherichia coli*, Bundesinstitut für Risikobewertung, Diedersdorfer Weg 1, D-12277 Berlin, Germany; 5 Centre for Paediatric Gastroenterology, Royal Free and University College Medical School, London, UK; 6 Fundación Caubet-Cimera, Recinto Hospital Joan March, Carretera Soller Km 1207110 Bunyola, Mallorca, Spain

## Abstract

Enteropathogenic *Escherichia coli* (EPEC) is a major cause of infantile diarrhoea in developing countries. While colonizing the gut mucosa, EPEC triggers extensive actin-polymerization activity at the site of intimate bacterial attachment, which is mediated by avid interaction between the outer-membrane adhesin intimin and the type III secretion system (T3SS) effector Tir. The prevailing dogma is that actin polymerization by EPEC is achieved following tyrosine phosphorylation of Tir, recruitment of Nck and activation of neuronal Wiskott–Aldrich syndrome protein (N-WASP). In closely related enterohaemorrhagic *E. coli* (EHEC) O157 : H7, actin polymerization is triggered following recruitment of the T3SS effector TccP/EspF_U_ (instead of Nck) and local activation of N-WASP. In addition to *tccP*, typical EHEC O157 : H7 harbour a pseudogene (*tccP2*). However, it has recently been found that atypical, sorbitol-fermenting EHEC O157 carries functional *tccP* and *tccP2* alleles. Interestingly, intact *tccP2* has been identified in the incomplete genome sequence of the prototype EPEC strain B171 (serotype O111 : H−), but it is missing from another prototype EPEC strain E2348/69 (O127 : H7). E2348/69 and B171 belong to two distinct evolutionary lineages of EPEC, termed EPEC 1 and EPEC 2, respectively. Here, it is reported that while both EPEC 1 and EPEC 2 triggered actin polymerization via the Nck pathway, *tccP2* was found in 26 of 27 (96.2 %) strains belonging to EPEC 2, and in none of the 34 strains belonging to EPEC 1. It was shown that TccP2 was: (i) translocated by the locus of enterocyte effacement-encoded T3SS; (ii) localized at the tip of the EPEC 2-induced actin-rich pedestals in infected HeLa cells and human intestinal *in vitro* organ cultures *ex vivo*; and (iii) essential for actin polymerization in infected Nck−/− cells. Therefore, unlike strains belonging to EPEC 1, strains belonging to EPEC 2 can trigger actin polymerization using both Nck and TccP2 actin-polymerization signalling cascades.

## INTRODUCTION

Enteropathogenic *Escherichia coli* (EPEC) is the leading cause of childhood diarrhoea in developing countries (reviewed by Chen & Frankel, 2005). EPEC strains belong to a large number of O : H serotypes (Trabulsi *et al.*, 2002) and are divided into typical and atypical categories (Kaper, 1996). Typical EPEC are defined by the presence of the locus of enterocyte effacement (LEE) pathogenicity island (McDaniel *et al.*, 1995), and the EPEC adherence factor (EAF) plasmid (Baldini *et al.*, 1983) that carries the transcriptional regulator locus *per* (Mellies *et al.*, 1999), and encodes the type IV bundle-forming pilus (BFP) (Girón *et al.*, 1991). The most common O serogroups of typical EPEC are O55, O111, O119, O127 and O142. Typical EPEC are further divided into two distinct evolutionary lineages known as EPEC 1 and EPEC 2 (Orskov *et al.*, 1990). The EPEC 1 branch is characterized by expression of flagella serotypes H6 and H34 (Whittam *et al.*, 1993), the presence of a complete *tra* region on the EAF plasmid (Brinkley *et al.*, 2006), and a distinctive antigenic type *α* of the outer-membrane adhesin intimin (Adu-Bobie *et al.*, 1998), while the EPEC 2 branch expresses the flagella serotype H2 (or H−) (Whittam *et al.*, 1993), lacks the *tra* region (Brinkley *et al.*, 2006), and expresses the intimin subtype *β* (Adu-Bobie *et al.*, 1998).

Enterohaemorrhagic *E. coli* (EHEC) is a subgroup of Verocytotoxin (VT)-producing *E. coli* (VTEC) that can cause bloody diarrhoea, haemorrhagic colitis and haemolytic–uraemic syndrome (HUS). *E. coli* O157 : H7 is the most common and virulent EHEC serotype that is implicated worldwide in human disease (reviewed by Karch *et al.*, 2005).

EPEC and EHEC colonize the gut mucosa via attaching and effacing (A/E) lesion formation, which is characterized by intimate attachment of the pathogen to the host intestinal epithelium, localized effacement of the brush border microvilli (Knutton *et al.*, 1987), and localized actin polymerization. A/E lesion formation and actin polymerization are dependent on the LEE-encoded type III secretion system (T3SS) (Jarvis *et al.*, 1995), the adhesin intimin (Jerse *et al.*, 1990), and translocation of the effector protein Tir (translocated intimin receptor) (Kenny *et al.*, 1997). Once translocated, Tir is integrated into the host-cell plasma membrane, in which it adopts a hairpin loop topology (Hartland *et al.*, 1999), with the extracellular loop presented above the plasma membrane acting as a receptor for the bacterial adhesin intimin (reviewed by Frankel *et al.*, 2001). Intimin-mediated clustering of Tir (Campellone *et al.*, 2004b) leads to accretion of several cytoskeletal proteins to the intracellular amino- and carboxy-terminal Tir domains, linking the extracellular bacterium to the host-cell cytoskeleton (Goosney *et al.*, 2001) and triggering actin remodelling into pedestal-like structures. Tir of the prototype EPEC O127 : H6 strain E2348/69, which belongs to EPEC 1, harbours a tyrosine residue Y474 that is present in the context of a consensus binding site for the mammalian adaptor protein Nck (Y_P_DEP/D/V) (Campellone *et al.*, 2002; Gruenheid *et al.*, 2001). Tyrosine phosphorylation of Tir [Tir_(Y-P)_] by host-cell kinases (Phillips *et al.*, 2004; Swimm *et al.*, 2004) recruits Nck to the site of bacterial attachment, which in turn binds the actin nucleation-promoting factor neuronal Wiskott–Aldrich syndrome protein (N-WASP), initiating actin polymerization via activation of the actin-related protein 2/3 (Arp2/3) complex (Lommel *et al.*, 2001). In contrast, the equivalent position in Tir EHEC O157 : H7 is occupied by serine [Tir_(S)_], and as such, Nck is not involved in actin polymerization by EHEC O157 : H7 (Gruenheid *et al.*, 2001). Instead, EHEC O157 : H7 requires a second bacterial T3SS effector protein, TccP (Tir-cytoskeleton coupling protein; also termed EspF_U_, because it shares 35 % identity with the T3SS effector EspF), which binds, recruits and activates N-WASP to trigger localized actin polymerization (Campellone *et al.*, 2004a; Garmendia *et al.*, 2004). The minimal region of Tir EHEC O157 that is needed for recruitment of TccP and induction of actin polymerization is a 12 aa motif at the C terminus (Campellone *et al.*, 2006; Allen-Vercoe *et al.*, 2006). This 12 aa motif is conserved in Tir EPEC O127 : H6, and has been implicated in an Nck-independent actin-remodelling pathway during infection with O127 : H6 EPEC (Campellone and Leong, 2005). Importantly, TccP does not bind Tir directly (Campellone *et al.*, 2004a; Garmendia *et al.*, 2004).

*tccP* is carried on prophage CP-933U/Sp14 (Campellone *et al.*, 2004a; Garmendia *et al.*, 2004) and consists of a unique 80 aa N-terminal region (involved in protein translocation) and several almost identical 47 aa proline-rich repeats (PRRs) (Garmendia *et al.*, 2006). In a recent survey of clinical and environmental strains, *tccP* was found in 100 % of EHEC O157 : H7 and in a minority of EPEC and non-O157 EHEC strains (Garmendia *et al.*, 2005). Of particular importance is the fact that in *tccP*-positive EPEC, Tir is tyrosine-phosphorylated [Tir_(Y-P)_] and simultaneously recruits Nck and TccP under attached bacteria during infection of cultured epithelial cells (Whale *et al.*, 2006).

EHEC O157 : H7 strains Sakai and EDL933 also contain pseudo *tccP* genes (*ECs1126* and *Z1385*, respectively, which have also been referred to as *espF_M_* by Campellone *et al.* 2004a), which are carried on prophage Sp4/CP-933M. A deletion of a single (T/A) base pair at position 28 introduces a translational frameshift and a premature stop codon. However, we have recently found that *β*-glucuronidase-positive/sorbitol-fermenting strains of EHEC O157 harbour an intact *ECs1126* gene, in addition to *tccP* (Ogura *et al.*, 2007). In order to discriminate between the *tccP* alleles, we named *ECs1126*, which is carried on prophage Sp4/CP-933M, *tccP2*. The aim of this study was to determine the distribution and function of *tccP2* in typical EPEC belonging to serogroups O55, O84, O111, O114, O127 and O142, which we have previously reported to be *tccP* gene-negative (Garmendia *et al.*, 2005) O119 : H6.

## METHODS

### PCR amplification of *tccP2* and *tir* and colony blot hybridization.

Clinical EPEC strains are listed in Table 1. Conventional PCR was used to amplify *tccP2* with gene-specific tccP2-F and tccP2-R primers. Forward, gene-specific primers tirY474-F and tirS478-F were used together with a conserved reverse primer (tir-R) to discriminate between *tir*_E2348/68_ and *tir*_Sakai_ gene types [that encode Tir_(Y–P)_ and Tir_(S)_, respectively]; primers used in this study are listed in Table 2. Colony blot hybridization was performed using standard protocols and a *tccp2* gene probe.

### Locus-specific sequencing.

The *tccP2* genes and their 5′- and 3′-flanking regions were amplified using a Blend taq PCR amplification Kit (Toyobo) and the PCR primer pairs tccP2-SFb and tccP2-SRb (Table 2). Direct sequencing of the PCR products was done using the primers used for amplification and an ABI PRISM 3100 automated sequencer. When necessary, internal sequencing primers were used.

### Preparation of TccP rabbit antiserum.

A PCR fragment encoding a truncated TccP_EDL933_ derivative comprising the unique N terminus and two PRRs (TccP_N2R_) was cloned into pET28-a as described previously (Ogura *et al.*, 2007); TccP_N2R_-His was purified as described by Hartland *et al.* (1999), and polyclonal TccP_N2R_-His antiserum was produced in rabbits at Covalab.

### Preparation of protein samples for detection of TccP and TccP2 by Western blotting.

Protein preparations from whole-cell extracts were dissolved in protein-denaturing buffer before PAGE and Western blotting. TccP was detected using a rabbit polyclonal anti-TccP primary antibody (diluted 1 : 1000) and porcine anti-rabbit IgG–horseradish peroxidase conjugate secondary antibody (Dako).

### Bacterial strains, growth conditions and plasmids.

Bacterial strains used for the functional analysis are listed in Table 3. Bacteria were grown at 37 °C, with aeration in Luria–Bertani (LB) medium or Dulbecco's Modified Eagle's Medium (DMEM), supplemented with ampicillin (100 μg ml^−1^) or kanamycin (50 μg ml^−1^), or both, when necessary. *E. coli* B171*ΔtccP2* mutant was constructed using the *λ* Red recombinase method (Datsenko & Wanner, 2000). Disruption of *tccP2* was performed with a kanamycin-resistance cassette generated with primers B171tccP-F1 and B171tccP-R1 using pKD4 as template. Purified PCR product was electroporated into *E. coli* B171(pKD46). Clones were grown on LB medium+kanamycin to select for kanamycin (Δ*tccP2*) resistance. pKD46 was cured from the resulting strains by growth at 43 °C. Primers flanking the deleted region and inside the kanamycin-resistance cassette were used in PCR to verify the deletion (primer pairs K1 and tccP2-F, and K2 and tccP2-R).

pICC364 is a derivative of pCX340 (Charpentier & Oswald, 2004). An 894 bp fragment containing *tccP2* was amplified by PCR from B171 genomic DNA using primers pCX-B171tccP-F1 and pCX-B171tccP-R1. The 915 bp PCR product, containing terminal *Eco*RI and *Not*I sites, was digested and ligated into pCX340, generating pICC364.

pICC365 and pICC366 are derivatives of pSA10 (Schlosser-Silverman *et al.*, 2000), a vector containing multiple cloning sites downstream of the *tac* promoter. An 894 bp fragment containing *tccP2* was amplified by PCR from B171 genomic DNA using primers pkk-tccP2-F1 and pkk-tccP2-R1. A 1176 bp fragment containing *tccP2* was amplified by PCR from EPEC O111 : H2 strain ICC215 genomic DNA using primers pkk-tccP2-F1 and pkk-tccP2-R1. The 970 bp (B171 *tccP2*) and 1252 (ICC215 *tccP2*) bp PCR products, containing terminal *Eco*RI and *Pst*I sites, were digested and ligated into pSA10, generating pICC365 and pICC366, respectively.

### Antibodies and reagents.

Anti-*E. coli* O157 : H7 goat polyclonal antibody (Fitzgerald Industries International) was diluted 1 : 500. EPEC ICC199 and B171 strains were detected with rabbit polyclonal Int280*β* antiserum (Adu-Bobie *et al.*, 1998), and EPEC E2348/69 was detected with rabbit polyclonal Int280*α* antiserum (Adu-Bobie *et al.*, 1998), both diluted 1 : 500. Rabbit polyclonal Tir_EPEC_ antiserum was diluted 1 : 500. Phosphotyrosine and Nck were detected using monoclonal mouse anti-phosphotyrosine clone 4G10 (Sigma) and rabbit polyclonal anti-Nck (Upstate) antibodies, diluted 1 : 250 and 1 : 150, respectively. Mouse anti-haemagglutinin (HA) mAb HA.11 (Covance) was diluted 1 : 200. Rhodamine-, Alexa 633- and Oregon Green-conjugated phalloidin (Invitrogen) were used at dilutions of 1 : 500, 1 : 500 and 1 : 100, respectively. Cy5-conjugated donkey anti-goat, rhodamine-conjugated donkey anti-goat, donkey anti-rabbit and Cy2-conjugated donkey anti-mouse antibodies (Jackson Immunoresearch Laboratories) were diluted 1 : 200. Samples were analysed using either a Zeiss LSM510 confocal laser scanning microscope or a Zeiss Axio Imager fluorescence microscope. Infected cells were fixed and fluorescent actin staining (FAS) used to determine the ability of each strain to trigger the formation of actin-rich pedestals. The efficiency of pedestal formation was quantified by measuring the percentage of adherent bacteria that associated with intense actin accretion. Randomly chosen cells harbouring discrete regions of 2–30 bacteria per cell were examined for each strain, and 300 bacteria, 50 per coverslip, from two or three independent infections carried out in duplicate, were counted in a blinded fashion. Statistical differences between groups were determined by ANOVA. To quantify the adherence of strains B171 and B171Δ*tccP2* to HeLa cells, the percentage of cells with at least three adhering bacteria was counted. For each strain, 200 randomly chosen cells from two independent infections carried out in duplicate were examined.

### Cell culture and infection.

HeLa cells (clone HtTA1) and mouse embryo fibroblast (MEF) N-WASP^−/−^, N-WASP^+/+^ (a kind gift from S. Snapper, Harvard Medical School, Boston, MA, USA), Nck1–/Nck2– and Nck1+/Nck2+ cell lines were grown in DMEM supplemented with 10 or 15 % fetal calf serum (FCS), respectively, containing 2 mM glutamine at 37 °C in 5 % CO_2_. Cells were seeded onto glass coverslips (12 mm diameter) in 24-well plates at a density of 5×10^4^ cells per well, 24 h before infection. Bacterial cultures grown in LB medium for 8 h were diluted 1 : 500 into DMEM and incubated as static cultures at 37 °C in 5 % CO_2_ overnight, and were used to infect cells for 3 h (EPEC strains) or 5 h (EHEC strains). For immunofluorescence, cell monolayers were fixed in 3.7 % paraformaldehyde in PBS, pH 7.4, for 15 min at room temperature, and washed three times with PBS. Antibodies were diluted in 10 % horse serum, 0.1 % saponin in PBS. Coverslips were washed twice in PBS containing 0.1 % saponin, incubated for 30 min with primary antibodies, washed twice with 0.1 % saponin in PBS, and incubated for 30 min with secondary antibodies. Coverslips were washed twice in 0.1 % saponin in PBS, once in PBS and once in water, and mounted with Aqua-Poly/Mount (Polysciences).

### Effector-protein translocation assay.

Overnight EPEC cultures were diluted 1 : 100 into 5 ml DMEM containing 10 % FCS and 2 mM glutamine, and incubated at 37 °C in 5 % CO_2_ for 3.5 h (preactivation). HeLa cells were infected with the preactivated bacteria for 30 min before IPTG was added at a final concentration of 1 mM, and the infection was allowed to proceed for an additional 1 h. Cell monolayers were washed three times with PBS, and covered with 100 μl PBS and 25 μl 6× concentrated *β*-lactamase substrate CCF2/AM (Charpentier and Oswald, 2004). The cells were incubated for 2.5 h at room temperature, washed three times with PBS, covered with coverslides, and live cells were observed under a Nikon Eclipse E600 fluorescence microscope using a UV-2A filter set (330–380 nm excitation). Pictures were taken under a Nikon digital camera DXM1200.

### Human intestinal *in vitro* organ culture (IVOC).

Tissue was obtained with fully informed parental consent and local ethical committee approval, using grasp forceps during routine endoscopic investigation of intestinal disorders. Small intestinal mucosal biopsies, which appeared macroscopically normal, were taken for organ-culture experiments as described by Hicks *et al.* (1998). Adherence of strains B171 and B171Δ*tccP2* was examined using tissue from two patients (aged 159 and 181 months) for scanning electron microscopy, and two further cases (aged 194 and 195 months) for cryosectioning and immunostaining. In each experiment, a non-infected sample was included to exclude endogenous bacterial adhesion. Samples for scanning electron microscopy were processed as described by Hicks *et al.* (1998). For immunofluorescence, samples were embedded in OCT compound (Sakura), snap-frozen in liquid nitrogen, and stored at −70 °C until use. Serial sections of 8 μm were cut with an MTE cryostat (SLEE Technik), picked up on poly-l-lysine-coated slides, and air-dried. Tissue sections were fixed in formalin for 10 min, and blocked with 0.5 % BSA and 2 % normal goat serum in PBS for 20 min at room temperature. Slides were incubated in primary antibody (rabbit anti-Nck, rabbit anti-TccP) for 60 min at room temperature, and washed and incubated in Alexa Fluor 488-conjugated goat anti-rabbit IgG (Molecular Probes) for 30 min. Counterstaining of bacteria and cell nuclei was performed using propidium iodide (Sigma). Epithelial cells were stained with mouse anti-cytokeratin (Dako) and Alexa Fluor 647-conjugated goat anti-mouse IgG (Molecular Probes). Sections were analysed with a Radiance 2100 confocal laser scanning microscope equipped with an argon–krypton laser and a red diode (Bio-Rad).

## RESULTS

### *tccP2* is absent from EPEC 1 and associated with the EPEC 2 lineage

**Fig. 1. f1:**
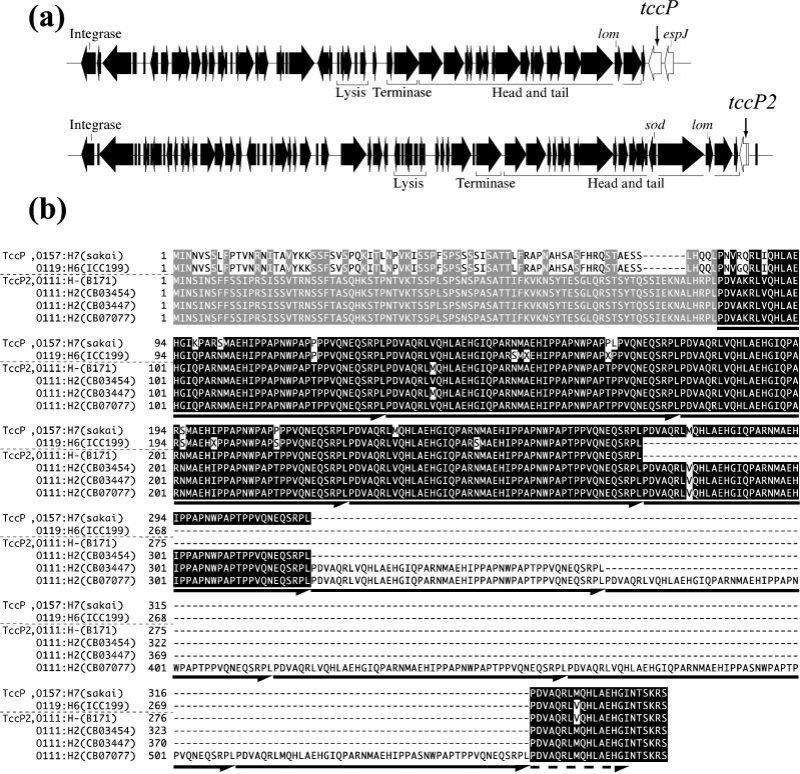
(a) Schematic representation of the genomic localization of *tccP* and *ECs1126* (renamed *tccP2*) in EHEC O157 : H7 (strain Sakai). *tccP* is located on prophage Sp14 (top) and *tccP2* on Sp4 (bottom). (b) Multiple sequence alignment of TccP of EHEC O157 : H7 (strain Sakai) and EPEC O119 : H6 (strain ICC199), and TccP2 of EPEC O111 : H− (strain B171) and EPEC O111 : H2 (strains CB03454, CB03447 and CB07077). The unique N terminus is shaded grey and the PRRs are shaded black. The complete unit of a PRR and a partial C-terminal repeat are indicated by arrows and a dashed arrow, respectively. The TccP sequence of ICC199 was taken from Whale *et al.* (2006) (accession no. DQ206456), and that for TccP2 of B171 from its unfinished genome sequence (AAJX01000044.1). Note that the *tccP2* gene is not annotated in the B171 genome sequence.

We employed conventional PCR to amplify *tccP2* (Fig. 1a[Fig f1]) using DNA from typical EPEC isolates as a template and gene-specific primers. *tccP2* was found in 26 of 27 (96.2 %) strains belonging to EPEC 2, but in none of the 34 EPEC 1 isolates (Table 1) and O119 : H6, which is evolutionarily distinct from EPEC 1 and EPEC 2 (Whittam & McGraw, 1996), and which we have previously shown encodes biologically active TccP (Whale *et al.*, 2006). The *tccP2* amplicons varied in length from 700 to 1800 bp (Table 1). We further confirmed the absence of *tccP* or *tccP2* in 14 randomly chosen, PCR-negative isolates by colony blot hybridization (data not shown).

Locus-specific PCR was used to amplify and sequence the *tccP2* locus of representative strains, confirming the presence of an intact ORF. Amino acid sequence alignment of the TccP2 polypeptides revealed that other than differences in the number of PRRs, which ranged from three to 10, the proteins shared a high level of sequence similarity (Fig. 1b[Fig f1]). Moreover, the PRRs of TccP2 overlapped almost exactly with those of TccP of EHEC O157 : H7 and EPEC O119 : H6 (Fig. 1b[Fig f1]).

### TccP2 is a translocated effector protein

**Fig. 2. f2:**
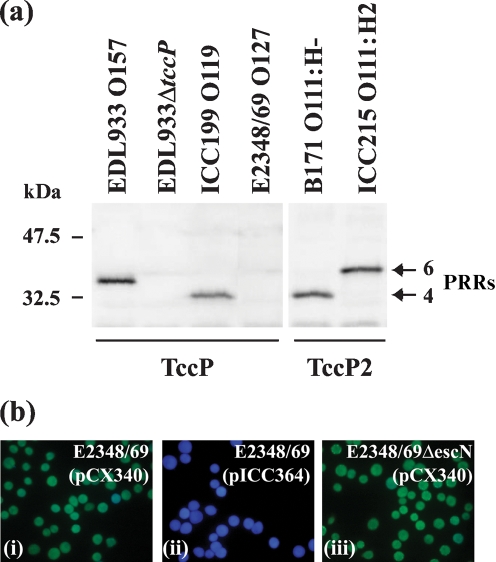
(a) TccP was detected with TccP antiserum in bacterial whole-cell lysates of EHEC EDL933 and EPEC ICC199, but not EDL933Δ*tccP* or EPEC E2348/69. TccP2 was also detected using TccP antiserum in lysates of EPEC O111 : H2 strain ICC215 and EPEC O111 : NM strain B171. (b) TccP2 is a T3SS-translocated effector. Translocation of the EPEC B171 effector protein TccP2 into live HeLa cells using TEM-1 fusion and fluorescence microscopy is shown. HeLa cells were infected with wild-type EPEC E2348/69 carrying pCX340 (negative control) (i), and E2384/69 (ii) and E2348/69Δ*escN* (iii) strains expressing TccP2__B171_–TEM fusion protein. *β*-Lactamase activity in HeLa cells was revealed by the blue fluorescence emitted by the cleaved CCF2 product (cells infected with E2348/69 expressing TccP2–TEM), whereas CCF2 emitted a green fluorescence (cells infected with Δ*escN* mutant expressing TccP2–TEM).

Antiserum raised against TccP is cross-reactive with TccP2 due to the sequence identity of the PRRs. Using the antiserum to analyse whole-cell extracts of *tccP*-negative/*tccP2*-positive EPEC 2 lineage strains B171 (O111 : H−) and ICC215 (O111 : H2), and *tccP*-positive/*tccP2*-negative strains EHEC EDL933 (O157 : H7) and EPEC ‘non-1 non-2 lineage’ ICC199 (O119 : H6) as controls, revealed reactive bands of different sizes that correlated with differences in the number of PRRs; no band was detected in the *tccP*-negative/*tccP2*-negative EPEC 1 strain E2348/69 (O127 : H6) or in EDL933Δ*tccP* (Fig. 2a[Fig f2]). These results show that *tccP2* is expressed in EPEC 2 strains.

TccP2 of EPEC 2 strain B171 is 77 % identical (87 % similar) to TccP of EHEC O157 : H7. However, while the PRRs were nearly identical, the N termini showed only 40 % identity (Fig. 1b[Fig f1]). Since this region of TccP contains the critical translocation signal (Garmendia *et al.*, 2006), we used the TEM-1 *β*-lactamase-based translocation assay (Charpentier & Oswald, 2004) to determine if TccP2__B171_ was translocated into the host cell. Translocation was detected directly within living host cells by using the fluorescent *β*-lactamase substrate CCF2/AM. HeLa cells were infected with wild-type and T3SS-deficient Δ*escN* mutant EPEC strains carrying pICC364, a plasmid that encodes a translational fusion of TccP2__B171_ to TEM-1. Expression of the fusion protein in these strains was verified by Western blot (data not shown), and translocation of the protein into infected HeLa cells was analysed (Fig. 2b[Fig f2]). Uninfected HeLa cells or cells infected with E2348/69(pCX340) (negative control, empty vector) appeared green, indicating the absence of TEM-1 activity (Fig. 2b[Fig f2], i). Cells infected with E2348/69(pICC364) expressing TccP2__B171_–TEM-1 appeared blue (Fig. 2b[Fig f2], ii), indicating that TEM-1 was translocated into the host cells. Moreover, this translocation was fully dependent on a functional T3SS, given that it was not observed when HeLa cells were infected with E2348/69Δ*escN* (pICC364) (Fig. 2b[Fig f2], iii). These results show that TccP2 is an effector protein translocated into host cells by the LEE-encoded T3SS.

### B171 triggers Nck-independent actin polymerization

**Fig. 3. f3:**
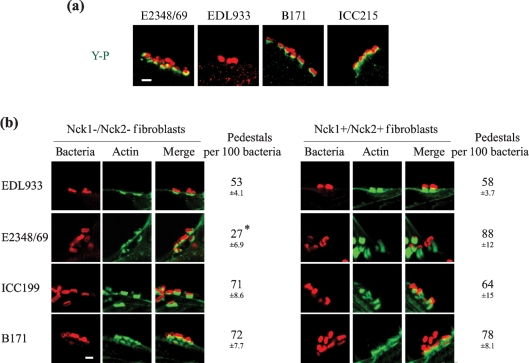
(a) Widespread Tir_(Y-P)_, labelled in green, was observed at the site of bacterial adhesion after infection of HeLa cells with strains E2348/69, B171 and ICC215, but not with EDL933. Bar, 2 μm. (b) FAS. EDL933, ICC199 and B171, but not E2348/69, triggered efficient actin polymerization following infection of Nck1–/Nck2– fibroblasts. All strains tested, including E2348/69, efficiently induced actin polymerization in infected Nck1+/Nck2+ fibroblasts. The asterisk indicates a statistically significant difference in the percentage of pedestals triggered on Nck1−/Nck2− fibroblasts, compared to that triggered on Nck1+/Nck2+ fibroblasts by EPEC E2348/69 (*P*<0.05). Bacteria, labelled in red, were detected with anti-intimin or anti-EHEC antibodies for EPEC E2348/69, ICC199 and B171 strains, and EHEC EDL933, respectively. Actin was labelled in green with Oregon Green-conjugated phalloidin. Bar, 2 μm.

Activation of the phospho-Tir [Tir(P)]–Nck actin-remodelling pathway is necessary for induction of actin-rich pedestals during infection with EPEC 1 strain E2348/69 (O127 : H7) (Campellone *et al.*, 2004b). In contrast, Nck is not recruited to the site of bacterial adhesion during infection with EHEC O157 : H7 (EDL933) (Gruenheid *et al.*, 2001), at which Tir and TccP are necessary for A/E lesion formation (Campellone *et al.*, 2004a; Garmendia *et al.*, 2004). In order to characterize TccP2-positive EPEC 2, HeLa cells were infected with strains B171 and ICC215. As controls, HeLa cells were infected with strains E2348/69 and EDL933. Immunostaining revealed tyrosine phosphorylation (a signal that previous studies have shown to correspond to Tir_(Y-P)_; Kenny *et al.*, 1997) below adherent E2348/69, B171 and ICC215, but not EDL933 (Fig. 3a[Fig f3]).

A feature of strains harbouring a functional *tccP* gene is their ability to efficiently trigger actin-pedestal formation on fibroblast cells lacking Nck (Gruenheid *et al.*, 2001; Campellone *et al.*, 2004a; Whale *et al.*, 2006). Accordingly, we compared the ability of EPEC 2, EPEC 1 and EHEC O157 : H7 to induce actin-pedestal assembly during infection of Nck1−/Nck2− and Nck1+/Nck2+ fibroblasts. Phalloidin staining and quantification of the efficiency of pedestal formation (see Methods) revealed that EDL933 was able to induce actin accretion during infection of an Nck1−/Nck2− fibroblast cell line, at a similar efficiency to infection of a control Nck1+/Nck2+ fibroblast cell line (Fig. 3b[Fig f3]). In agreement with Campellone & Leong, 2005, strain E2348/69 induced less intense actin accretion at significantly reduced frequency in Nck1−/Nck2– fibroblast cells in comparison to Nck1+/Nck2+ cells (*P*<0.05), when preactivated by static growth in tissue-culture medium and 5 % CO_2_ (Fig. 3b[Fig f3]). The residual and inefficient ability of EPEC E2348/69 to trigger actin polymerization is likely to be due to a recently identified Nck-independent pathway mediated by unknown cellular or bacterial factors (Campellone & Leong, 2005). In contrast, in a similar manner to TccP-expressing EPEC O119 : H6 strain ICC199 and EHEC O157 : H7 strain EDL933, TccP2-expressing B171 was able to trigger actin polymerization during infection of an Nck1−/Nck2− fibroblast cell line, at a similar efficiency as during infection of an Nck1+/Nck2+ fibroblast cell line (Fig. 3b[Fig f3]). Thus, it appears that B171 is able to utilize an Nck-independent pathway to efficiently induce actin polymerization upon infection.

### Functional analysis of TccP2

**Fig. 4. f4:**
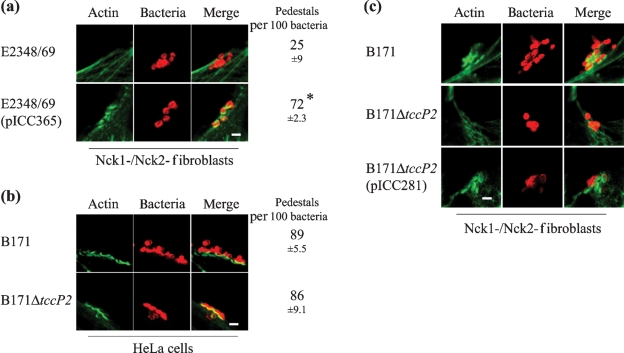
(a) Nck1−/Nck2− fibroblasts were infected with *tccP*^−^/*tccP2*^−^ EPEC 1 strain E2348/69 and E2348/69(pICC365-*tccP2*__B171_). Expression of TccP2__B171_ compensated for the absence of Nck, enabling efficient and significantly different (*P*<0.05) production of actin-rich pedestals at the site of bacterial attachment. Bar, 2 μm. (b) B171 and its isogenic B171Δ*tccP2* mutant strain induced actin polymerization at a similar efficiency in infected HeLa cells. Bar, 2 μm. (c) Efficient actin polymerization was detected following infection of Nck1−/Nck2− fibroblasts with B171, but not with B171Δ*tccP2*. Wild-type phenotype was restored by complementation of B171Δ*tccP2* with pICC281 (*tccP*) or pICC365 (*tccP2*) (data not shown). Bacteria, labelled in red, were detected with anti-intimin *β* or anti-intimin α antibodies for B171 and E2348/69, respectively. Actin was labelled in green with phalloidin–Oregon Green. Bar, 2 μm.

In order to elucidate the function of TccP2, we carried out infections of Nck1–/Nck2– fibroblast cell lines with EPEC 1 E2348/69 expressing *tccP2*__B171_ (pICC365). Phalloidin staining and quantification of efficiency of pedestal formation revealed that expression of *tccP2* in E2348/69 significantly enhanced its ability to trigger actin polymerization in Nck1–/Nck2– cells (Fig. 4a[Fig f4]), suggesting that translocated TccP2 is able to promote host-cell actin polymerization under adherent E2348/69 in the absence of a functional Tir(P)–Nck actin-remodelling pathway.

In order to determine the role of *tccP2* in B171-induced A/E lesions, a non-polar deletion of *tccP2* was generated, producing strain B171Δ*tccP2* (ICC216). Infection of HeLa cells revealed that B171Δ*tccP2* induced actin polymerization under attached bacteria in a similar manner to wild-type B171 (Fig. 4b[Fig f4]), despite exhibiting reduced cell adherence (B171Δ*tccP2* adhered to 48±13 % of cells; in comparison, wild-type B171 adhered to 99.5±1 % of cells). However, B171Δ*tccP2* was unable to trigger actin polymerization beneath adherent bacteria during infection of Nck1−/Nck2− fibroblasts (Fig. 4c[Fig f4]). Similar to published observations regarding inefficiency of binding and effector translocation into Nck-deficient MEF cell lines (Campellone *et al.*, 2004a), B171Δ*tccP2* interacted with Nck-deficient fibroblasts at levels too low to allow strict quantification of pedestal formation. The ability of B171Δ*tccP2* to trigger actin accretion was completely restored by introduction of plasmids encoding TccP__EDL933_ (pICC281) (Fig. 4c[Fig f4]) and TccP2__B171_ (pICC365) (data not shown). These results suggest that B171, similar to EHEC O157, is able to trigger localized actin polymerization in an Nck-independent and TccP-dependent manner. Significantly though, B171 is different from EHEC O157, as it can also trigger actin polymerization via a TccP-independent mechanism.

**Fig. 5. f5:**
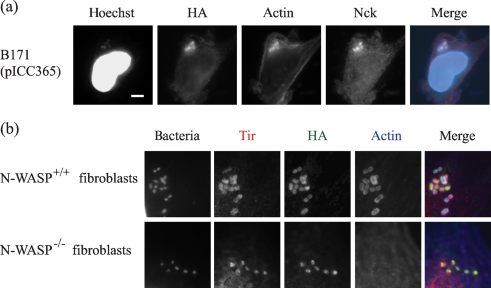
(a) Nck and TccP2 were simultaneously recruited and co-localized at the site of strain B171(pICC365-*tccP2*__B171_)-induced actin assembly beneath adherent bacteria during infection of HeLa cells. Nck was labelled in green using an anti-Nck antibody, TccP2–HA was labelled in far red with an anti-HA mAb, and actin was labelled in red, using rhodamine-conjugated phalloidin. Bacteria and cell nuclei were visualized with Hoechst stain (blue). Separate monochrome images of the UV, far-red, red and green fluorescence channels are shown, as well as merged images of all channels (right column). Bar, 10 μm. (b) Tir and TccP2 co-localized at the site of B171Δ*tccP2*(pICC365-*tccP2*__B171_) attachment during infection of N-WASP^+/+^ and N-WASP^−/−^ fibroblasts. However, induced actin assembly was only detected beneath adherent bacteria during infection of N-WASP^+/+^ fibroblasts. Tir was labelled in red, TccP2–HA was labelled in green, and actin was labelled in far red (shown in blue). Bacteria were visualized with Hoechst stain (shown in monochrome).

In order to determine whether both the Nck pathway and the TccP pathway are activated at the site of bacterial attachment, pICC365 encoding C-terminal HA-tagged TccP2__B171_ was introduced into B171. Co-immunostaining with HA and Nck antibodies revealed that Nck and TccP2 were simultaneously concentrated in actin-rich pedestals beneath adherent bacteria (Fig. 5a[Fig f5]), indicating that B171 has the ability to simultaneously utilize the Nck- and TccP2-mediated actin-remodelling pathways. To confirm that TccP2 functions upstream of N-WASP in the actin-polymerization cascade, we infected an N-WASP-deficient fibroblast cell line (N-WASP^−/−^ MEFs with B171Δ*tccP2*(pICC365) (expressing HA-tagged TccP2). Co-immunostaining of infected cells with Tir_EPEC_ antiserum and HA antibodies revealed that both Tir and TccP2-HA were recruited to the site of bacterial attachment in the absence of N-WASP (Fig. 5b[Fig f5]). As expected, no TccP-HA was detected beneath B171Δ*tccP2* (data not shown), and neither strain was able to trigger formation of actin pedestals on N-WASP^−/−^ MEFs. Infection and immunostaining of control N-WASP-proficient cells (N-WASP^+/+^ MEFs) revealed that Tir and TccP2 were recruited to sites of adherent B171Δ*tccP2* expressing HA-tagged TccP2, but crucially, actin pedestals were triggered. Taken together, these data show that TccP2 is recruited in the absence of N-WASP, and suggest that N-WASP is not required for the indirect interaction between Tir and TccP2, and that N-WASP is a critical factor in the B171-induced actin-polymerization cascade.

### Recruitment of TccP2 and Nck during infection of human intestinal biopsy samples with B171

**Fig. 6. f6:**
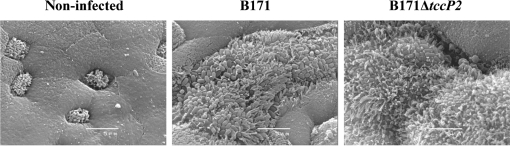
Both wild-type and Δ*tccP2* B171 strains induced A/E lesions in intestinal IVOC. Scanning electron micrographs of duodenal mucosa infected with B171 and B171Δ*tccP2* are shown*.* A non-infected sample was included as a negative control. Bars, 5 μm.

**Fig. 7. f7:**
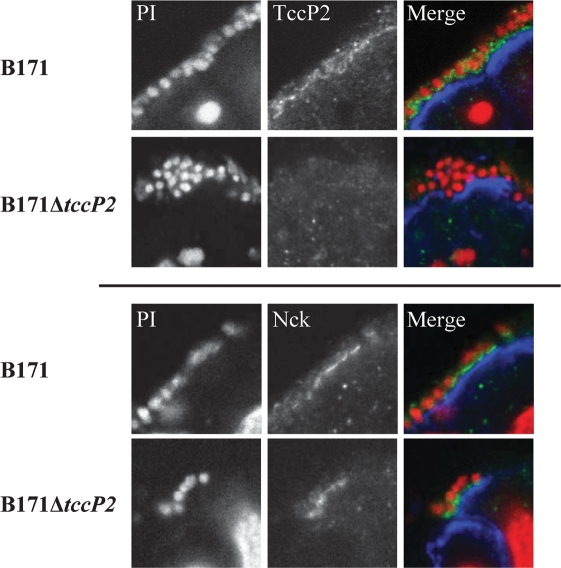
Nck was recruited to the site of IVOC adhesion of strains B171 and B171Δ*tccP2*, while TccP2 was found only under adherent B171. Terminal ileal mucosa was infected with B171 and B171Δ*tccP2* for 8 h, and cryosections were processed for immunofluorescence. Staining was performed for TccP2 (green in merged image, upper two panels) or Nck (green, lower two panels). Bacteria and cell nuclei were visualized by propidium iodide (PI) stain (red). Epithelial cells were counterstained with anti-cytokeratin (labelled blue). Separate monochrome images of the red and green fluorescence channels (left and middle column respectively) are shown, as well as merged images of all channels (right column).

To investigate the role of TccP2 during IVOC, paediatric small intestinal biopsy samples were infected with strain B171 and its isogenic *tccP2* mutant (B171Δ*tccP2*). As shown in Fig. 6, both wild-type and deletion mutant attached intimately to human intestinal mucosa, causing microvillus elongation in between adhering bacteria. Immunofluorescence staining of cryosectioned organ-culture samples showed that TccP2 was translocated into human intestinal epithelium and localized beneath adherent B171 bacteria. The host adaptor protein Nck was recruited by both wild-type and B171Δ*tccP2* bacteria; in contrast, and as expected, no TccP2 staining was observed in B171Δ*tccP2*-infected samples (Fig. 7).

### *tccP* and *tccP2* are functionally interchangeable

**Fig. 8. f8:**
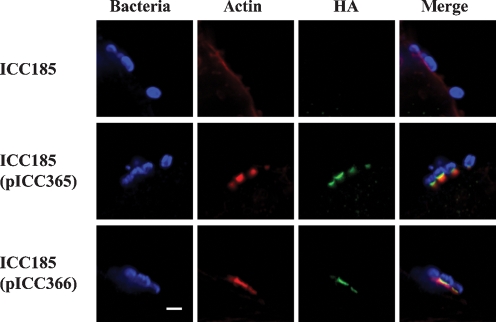
FAS and recruitment of TccP2 to the site of bacterial adhesion. HeLa cells were infected with strain EDL933Δ*tccP* for 5 h. Following fixation and permeabilization, bacteria were labelled in blue. Actin was detected by rhodamine–phalloidin, and TccP2–HA was labelled in green. Strain ICC185, unable to form actin-rich pedestals during infection, was complemented by both TccP2__B171_ (pICC365) and TccP2__ICC215_ (pICC366). TccP2 was detected beneath adherent ICC185(pICC365) and ICC185(pICC366), but not ICC185, and co-localized with polymerized actin at the tip of the triggered actin pedestal. Bar, 2 μm.

Considering that TccP__ICC199_ was 77 % identical to TccP2__B171_ and that TccP__EDL933_ complemented B171Δ*tccP2* (Fig. 4c[Fig f4]), we carried out a reciprocal experiment to determine the ability of TccP2 to complement an EDL933Δ*tccP* mutant strain. To this end, *tccP2* from strains B171 (consisting of four PRRs) and ICC215 (consisting of six PRRs), cloned under the control of an IPTG-inducible promoter and tagged with a C-terminal HA epitope (pICC365 and pICC366, respectively), was introduced into strain ICC185 (EDL933Δ*tccP*). The ability to complement the *tccP* mutation and induce actin-pedestal assembly during infection was analysed by immunofluorescence. Phalloidin staining revealed that *tccP2*__B171_ and *tccP2*__ICC215_ complemented the ability of EDL933Δ*tccP* to generate A/E lesions following infection of HeLa cells (Fig. 8). Co-staining of infected HeLa cells with an anti-HA mAb and phalloidin revealed that TccP2–HA was detected beneath ICC185(pICC365) and ICC185(pICC366) bacteria, co-localizing with F-actin at the tip of the pedestals (Fig. 8). In contrast, introduction of a plasmid-borne copy of *Z1385* (pseudo *tccP2* allele of EDL933) did not restore the ability of ICC185 to induce actin pedestals (data not shown). These data indicate that *tccP2* encodes a protein that can functionally substitute for TccP__EDL933_, and that both *tccP* homologues, *tccP* and *tccP2*, are functionally interchangeable.

## DISCUSSION

Until recently, the prevalent dogma concerning EPEC- and EHEC-triggered localized actin polymerization, based on studies of two prototypical strains (O127 : H6 EPEC 1 strain E2348/69 and O157 : H7 EHEC strain EDL933), was that formation of actin-rich pedestals is achieved via distinct signal-transduction pathways. A C-terminal 12 aa motif (including phosphorylated Y474) of Tir_EPEC_ binds Nck, which in turn recruits and activates N-WASP beneath adherent bacteria (Campellone et al., 2004b). N-WASP then recruits the Arp2/3 complex, leading to the generation of a network of actin filaments under attached bacteria. In contrast, a different C-terminal 12 aa motif of Tir_EHEC_O157_ (encompassing Y458) (Campellone *et al.*, 2006; Allen-Vercoe *et al.*, 2006) clusters TccP, which leads to the formation of actin-rich pedestals by an Nck-independent mechanism (Gruenheid *et al.*, 2001). However, through the analysis of a large number of clinical and environmental non-O157 EHEC and EPEC isolates, we have identified a subset of strains that have the potential to induce actin polymerization in the host eukaryotic cell by simultaneously utilizing the Tir(P)–Nck and Tir–TccP pathways (Garmendia *et al.*, 2005). The predominant EPEC group in this category are strains belonging to EPEC serotype O119 : H6 (Whale *et al.*, 2006), which is situated in the evolutionary tree in between the EPEC 1 and EPEC 2 lineages (Whittam & McGraw, 1996).

EPEC 1 strains are characterized by expression of flagellar antigens H6 or H34 (Whittam *et al.*, 1993), possession of a complete *tra* region (Brinkley *et al.*, 2006), and intimin *α*. In this study, we have shown that other characteristics of EPEC 1 strains are expression of Tir_(Y-P)_ and lack of *tccP* and *tccP2*. Importantly, strains belonging to O119 : H6 are unique, as they do not belong to EPEC 1 (Whittam & McGraw, 1996), express intimin type *β* (Adu-Bobie *et al.*, 1998), almost harbour *tccP* (Garmendia *et al.*, 2005), and may have a complete *tra* region (Brinkley *et al.*, 2006). These characteristics suggest that the evolution of this serotype followed a distinct path, through which it acquired virulence determinants horizontally. The EPEC 2 lineage is characterized by expression of flagellar antigens H2 or H− (Whittam *et al.*, 1993), intimin *β* (Adu-Bobie *et al.*, 1998) and Tir_(Y-P)_. Unexpectedly, we found that with the exception of only one isolate, all of the EPEC 2 strains tested contained intact *tccP2*. Sequence analysis of TccP2 from different isolates showed that, other than variation in the number of PRRs, the protein sequences were identical. The identification of TccP2 in prototypic EPEC strain B171 highlights the fortuitous nature of studying pathogenesis in prototypical strains, as the commonly used E2348/69, which is *tccP*- and *tccP2*-negative, allowed the identification of the role of Nck in A/E lesion formation.

Using B171 as a representative of EPEC 2 *tccP2*-expressing strains, we have shown that TccP2 is a T3SS-translocated effector involved in triggering actin remodelling during infection. In a similar manner to *tccP*-positive EPEC and EHEC strains, but in contrast to prototypical EPEC 1 strains, B171 was able to efficiently trigger Nck-independent actin polymerization, an activity dependent on *tccP2*. Due to the high level of sequence conservation between TccP and TccP2, we observed functional redundancy between the two TccP homologues: TccP2 was able to restore actin-polymerization ability to EDL933Δ*tccP* during infection of HeLa cells, and TccP was able to complement B171Δ*tccP2* for triggering actin remodelling during infection of Nck1−/Nck2− fibroblasts. Nevertheless, due to the difference between the N termini of TccP and TccP2, we cannot exclude the possibility of subtle functional differences.

**Fig. 9. f9:**
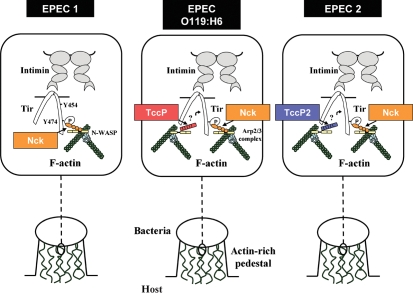
Summary of actin-polymerization cascades induced by EPEC 1, EPEC 2 and EPEC O119 : H6. All EPEC lineages translocate Tir that once inserted into the host-cell membrane serves as a receptor for the bacterial adhesin intimin. Intimin-mediated clustering of Tir triggers phosphorylation of tyrosine residue Y474 and concurrent recruitment of Nck. Nck recruits and activates N-WASP, leading to Arp2/3-dependent actin polymerization and pedestal formation. EPEC 2 and ‘non-1 non-2 lineage’ O119 : H6 EPEC, but not EPEC 1 bacteria, also translocate TccP2 and TccP, respectively, and are capable of recruiting N-WASP directly and triggering Nck-independent actin-pedestal formation. Note that TccP does not bind Tir directly, but via an unidentified host-encoded adaptor (indicated by a question mark).

In a similar way to TccP-positive EPEC strains, TccP2 is localized at the tip of the pedestal and co-localizes with Nck during B171 infection of epithelial cells. Of note, Tir_B171_ harbours both a Y474 equivalent in the context of a consensus Nck binding site, and a second tyrosine residue within a region that shares 75 % amino acid identity with a motif responsible for TccP/EspF_U_ recruitment in Tir_EHEC_ (Campellone *et al.* 2006). Moreover, both TccP2 and Nck are recruited to the site of B171 adhesion to human intestinal IVOC. In the absence of TccP2 (i.e. during infection with B171Δ*tccP2*), Nck was still recruited, suggesting that recruitment of Nck, bacterial adhesion and A/E lesion formation are not dependent on TccP2. A model describing the actin-polymerization cascades induced by EPEC 1, EPEC 2 and ‘non-1 non-2’ EPEC O119 : H6 is shown in Fig. 9. The ability of strains belonging to EPEC 2 to trigger actin polymerization using apparently redundant mechanisms raises intriguing questions regarding the possible spatial and temporal specificities of their function.

We have shown that possession of *tccP2* and Tir_(Y-P)_ is a characteristic of EPEC 2. This observation raises some interesting questions, including whether or not the ability to use both the Nck and TccP2 pathways confers an advantage upon EPEC 2, and if so, in which environments EPEC 2 strains are more virulent than EPEC 1 strains. Finally, strain E2348/69 (O127 : H7), which has been used worldwide as a prototype EPEC strain, harbours Tir_(Y-P)_ but is *tccP* negative. As such, when studying actin-pedestal formation, E2348/69 should no longer be used as a general representative of typical EPEC; it should now only be considered representative of EPEC 1, while B171 should be used as a prototype strain representing the EPEC 2 lineage.

## Figures and Tables

**Table 1. t1:** Distribution of *tccP2* among clinical EPEC 1 and EPEC 2 isolates nd, Not determined.

**Serotype**	**No. of strains**	***tccP2***	**Tir type**
**EPEC 1**			
O55 : H6	10	−	Y-P (5)*, nd (5)
O86 : H34	4	−	Y-P (3), nd (1)
O127 : H6	5	−	Y-P (3), nd (2)
O142 : H6	6	−	Y-P (4), nd (2)
O142 : H34	3	−	Y-P
**EPEC 2**			
O111 : H−	8	+[1150 bp]† (6)*, +[700 bp] (1), − (1)	Y-P
O111 : H2	18	+[1150 bp] (14), +[1000 bp] (3), +[1800 bp] (1)	Y-P
O114 : H2	1	+[1150 bp]	Y-P
**EPEC non-1 non-2**			
O119 : H6‡	6	−	Y-P

*The number of strains is given in parentheses. †The size of *tccP2* amplicons is indicated in brackets. ‡*tccP*-positive strain.

**Table 2. t2:** Primers used in this study

**Primer**	**Nucleotide sequence (5′–3′)**
tccP2-F	ATGATAAATAGCATTAATTCTTT
tccP2-R	TCACGAGCGCTTAGATGTATTAAT
tirY474-F	CATATTTATGATGAGGTCGCTC
tirS478-F	TCTGTTCAGAATATGGGGAATA
tir-R	TAAAAGTTCAGATCTTGATGACAT
tccP2-SFb	GGTAGATTTCATGCAAACGG
tccP2-SRb	AATAACCGGTAACTGTCAGGTC
B171tccP-F1	CACAGCACAAAAGCACACCTAACACGGTAAAAACCAGCTCACCTCTTTCTCGTGTAGGCTGGAGCTGCTTC
B171tccP-R1	GAGGTCTTGATTGTTCATTTTGTACTGGCGGCGTTGGCGGAGGCCAGTTACATATGAATATCCTCCTTAG
k1	CAGTCATAGCCGAATAGCCT
k2	CGGTGCCCTGAATGAACTGC
pkk-tccP2-F1	CCGGAATTCATGATAAATAGCATTAATTCTTTT
pkk-tccP2-R1	AAAACTGCAGTCAAGCGTAGTCTGGGACGTCGTATGGGTAAGCGTAGTCTGGGACGTCGTATGGGTACGAGCGCTTAGATGTATTAATGCC
pCX-B171tccP-F1	GGGTTTCATATGATAAATAGCATTAATTCTTTT
pCX-B171tccP-R1	CCGGAATTCTCCGAGCGCTTAGATGTATTAATG

**Table 3. t3:** *E. coli* strains and plasmids

**Strain or plasmid**	**Description**	**Source**
**Strains**		
EDL933	EHEC O157 : H7 *stx^−^*	ATCC*
E2348/69	Wild-type EPEC 1 O127 : H6	Levine *et al.* (1978)
ICC192	Δ*escN* : : Km in *E. coli* O127 : H6 E2348/69	Garmendia *et al.* (2004)
ICC199	Human clinical isolate *E. coli* O119 : H6 *tccP^+^* (non-1 non-2 EPEC)	Whale *et al.* (2006)
ICC215	Human clinical isolate *E. coli* O111 : H2 *tccP2^+^* (EPEC 2)	This study
B171	Wild-type EPEC 2 O111 : H− *tccP*2*^+^*	Riley *et al.* (1987)
ICC216	Δ*tccP2* : : Km in *E. coli* O111 : H− strain B171	This study
ICC185	Δ*tccP* : : Km in *E. coli* O157 : H7 EDL933	Garmendia *et al.* (2004)
**Plasmids**		
pCX340	pBR322 derivative used to generate *blaM* gene fusions	Charpentier & Oswald (2004)
pICC364	pCX340 derivative encoding TccP2__B171_ fused to TEM-1	This study
pKD46	Helper plasmid	Datsenko & Wanner (2000)
pSA10	pKK177-3 derivative containing *lac^q^*	Schlosser-Silverman *et al.* (2000)
pICC281	pSA10 derivative encoding TccP__O157_–FLAG fusion protein	Garmendia *et al.* (2004)
pICC365	pSA10 derivative encoding TccP2__B171_–HA	This study
pICC366	pSA10 derivative encoding TccP2__ICC215_–HA	This study

*American Type Culture Collection.

## Abbreviations

- A/E, attaching and effacing
- BFP, bundle-forming pilus
- EAF, enteropathogenic *Escherichia coli* adherence factor
- EHEC, enterohaemorrhagic *Escherichia coli*
- EPEC, enteropathogenic *Escherichia coli*
- FAS, fluorescent actin staining
- HA, haemagglutinin
- IVOC, *in vitro* organ culture
- LEE, locus of enterocyte effacement
- MEF, mouse embryo fibroblast
- PRR, proline-rich repeat
- N-WASP, neuronal Wiskott–Aldrich syndrome protein
- T3SS, type III secretion system
